# Impact of eIF4E phosphorylation at Ser209 via MNK2a on tumour recurrence after curative surgery in localized clear cell renal cell carcinoma

**DOI:** 10.18632/oncotarget.27017

**Published:** 2019-06-18

**Authors:** Osamu Ichiyanagi, Hiromi Ito, Sei Naito, Takanobu Kabasawa, Hidenori Kanno, Takafumi Narisawa, Masaki Ushijima, Yuta Kurota, Michinobu Ozawa, Toshihiko Sakurai, Hayato Nishida, Tomoyuki Kato, Mitsunori Yamakawa, Norihiko Tsuchiya

**Affiliations:** ^1^ Department of Urology, Yamagata University Faculty of Medicine, Yamagata, Japan; ^2^ Department of Urology, Yamagata Prefectural Kahoku Hospital, Kahoku, Japan; ^3^ Department of Pathological Diagnostics, Yamagata University Faculty of Medicine, Yamagata, Japan

**Keywords:** renal cell carcinoma, phospho-eIF4E (p-eIF4E), MNK (MAPK-interacting kinase), recurrence-free, epithelial−mesenchymal transition

## Abstract

**Background:** We investigated the roles of eIF4E phosphorylation (Ser209) in tumour recurrence after curative nephrectomy for localized clear cell renal cell carcinoma (ccRCC).

**Methods:** Expression of eIF4E, p eIF4E and MNKs (MAPK interacting kinases), was evaluated in surgical specimens obtained from consecutive non metastatic ccRCC patients (*n* = 290) by immunohistochemistry (IHC), immunoblotting, and qRT PCR at the protein and mRNA levels. In human RCC cell lines, the effects of eIF4E phosphorylation were examined using immunoblotting, proliferation, migration and invasion assays with pharmacological inhibitors (CGP57380 or ETP45835) and specific small interfering (si) RNAs against MNK1/2(a/b).

**Results:** In postoperative follow-up (median, 7.9 y), 40 patients experienced metastatic recurrence. In multivariate Cox analyses, higher IHC expression of p eIF4E in ccRCC significantly predicted a longer recurrence-free interval. eIF4E is phosphorylated mainly by MNK2a in tumour specimens and cell lines. In 786-O and A-498 cell lines, pharmacological inhibition of MNKs decreased p-eIF4E and increased vimentin and N cadherin but did not influence proliferation. Similarly, MNK2 or MNK2a inhibition with siRNA reduced p-eIF4E and enhanced vimentin translation, cell migration and invasion in the cell lines.

**Conclusions:** MNK2a-induced eIF4E phosphorylation may suppress metastatic recurrence of ccRCC, partially due to vimentin downregulation at the translational level, consequently leading to inhibition of epithelial–mesenchymal transition.

## INTRODUCTION

Renal cell carcinoma (RCC) is one of the most common malignant tumours originating in the kidney. Clear cell histology consists of approximately 80% of RCC [1, 2]. The incidence and mortality rates of RCC are increasing all over the world including Japan [1, 2]. An estimated 20%–30% of patients presented with metastatic disease during initial diagnosis [1, 2]. The survival rates for metastatic RCC have been drastically improved owing to the development of targeted drugs and immune checkpoint inhibitors [3]. However, the prognosis of patients with advanced-stage (T3-4, N+, M+) RCC remains extremely poor, with a 5-year overall survival (OS) rate of 10%–30% [1]. In contrast, the 5-year recurrence-free and overall survival rates of RCC at stage I were around 95%, respectively [1]. Up to 30% of patients who undergo curative surgery for clinically-confined RCC suffer from disease recurrence [4].

The translation initiation factor eukaryotic initiation factor (eIF) 4E plays a pivotal role in protein biosynthesis at a subcellular level [5]. eIF4E is released from eIF4E-binding protein 1 (4EBP1) that is phosphorylated by activation of mammalian target of rapamycin complex 1 (mTORC1). eIF4E is the rate-limiting component in binding and cap-dependent translation of certain mRNAs. In normal cells, mRNA and protein expression of eIF4E are regulated at relatively low levels and eIF4E expression and activity depends upon various signals and stresses from outside the cells [5]. In malignant cells, eIF4E is overexpressed to promote neoplastic transformation and tumorigenesis [5, 6]. eIF4E is persistently hyperactivated due to genetic alterations of pathway components located upstream of the 4EBP1/eIF4E axis, as the genes encoding 4EBP1 and eIF4E remain normal in the vast majority of cancers, including RCC [5, 7, 8].

Interacting with the scaffold eIF4G, eIF4E is phosphorylated at Ser209 by mitogen-activated protein kinase (MAPK) -interacting kinase 1/2 (MNK1/2) [9]. Human MNK1/2 are encoded by two different genes, *MKNK1* and *MKNK2*. The transcripts of *MKNK1* and *MKNK2* can be alternatively spliced, giving rise to two proteins, MNK1a/2a (the longer forms) and MNK1b/2b (the shorter forms) with functional differences in their N- and C-terminal regions [9]. No difference in the ability to phosphorylate eIF4E has been reported among MNK1/2/a/b isoforms [9]. However, the oncological roles of p-eIF4E in malignancies are not completely understood [10]. Herein, we aim to investigate: (1) the clinical relevance of p-eIF4E in predicting tumour recurrence after curable resection of localized ccRCC, and (2) the effects of p-eIF4E on tumour behaviours by inhibiting MNKs in RCC cell lines.

## RESULTS

### Clinicopathological backgrounds of ccRCC patients

Consecutive patients (*n =* 290) who underwent curative surgery for localized ccRCC were recruited in the present study. The median follow-up duration was 7.9 years (0.02–19.9 years) after radical (*n =* 186) or partial (*n =* 104) nephrectomy. Surgical approaches used were open (*n =* 178) or laparoscopic (*n =* 112). Patient demographics are summarised in Table 1. In the course of postoperative follow-up, forty patients experienced cancer recurrence in the lung, lymph nodes, liver, bone, adrenal glands, and other sites (*n =* 25, 10, 5, 5, 2, and 7, respectively). Local recurrence occurred in two cases. Eight patients presented with recurrent lesions in multiple organs. Of patients who had a recurrence, 21 died of ccRCC in the follow-up period.

**Table 1:** Clinicopathological backgrounds of study patients, and associations with eIF4E and p-eIF4E expression

Factors		All patients	eIF4E expression			p-eIF4E(S209) expression		
			Low	High	*P*	Low	High	*P*
Number of patients		290	104	186		142	148	
Age at nephrectomy, years	Mean ± SD [range]	62.8 ± 11.6 [28–85]	63.5 ± 11.6 [30–83]	62.4 ± 11.6 [28–85]	0.437^¶^	63.0 ± 11.6 [28–83]	62.6 ± 11.6 [34–85]	0.801^¶^
Sex	Male	192 (66.2)	68 (65.4)	124 (66.7)	0.897^#^	92 (64.8)	100 (67.6)	0.622^#^
	Female	98 (33.8)	36 (34.6)	62 (33.3)		50 (35.2)	48 (32.4)	
Tumour laterality	Left	136 (46.9)	52 (50.0)	84 (45.2)	0.463^#^	72 (50.7)	64 (43.2)	0.239^#^
	Right	154 (53.1)	52 (50.0)	102 (54.8)		70 (49.3)	84 (56.8)	
Pathological T stage	pT1a	169 (58.3)	61 (58.7)	108 (58.1)	0.692^§^	71 (50.0)	98 (66.2)	<0.001^§^
	pT1b	58 (20.0)	21 (20.2)	37 (19.9)		42 (29.6)	16 (10.8)	
	pT2	19 (6.6)	9 (8.7)	10 (5.4)		6 (4.2)	13 (8.8)	
	pT3	42 (14.5)	13 (12.5)	29 (15.6)		22 (15.5)	20 (13.5)	
	pT4	2 (0.7)	0 (0.0)	2 (1.1)		1 (0.7)	1 (0.7)	
Pathological N stage	pN0/NX	284 (97.9)	103 (99.0)	181 (97.3)	0.425^§^	142 (100.0)	142 (95.9)	0.030^§^
	pN1	6 (2.1)	1 (1.0)	5 (2.7)		0 (0.0)	6 (4.1)	
Fuhrman grade	G1	115 (39.7)	48 (46.2)	67 (36.0)	0.255^§^	61 (43.0)	54 (36.5)	0.614^§^
	G2	120 (41.4)	40 (38.5)	80 (43.0)		57 (40.1)	63 (42.6)	
	G3	39 (13.4)	13 (12.5)	26 (14.0)		18 (12.7)	21 (14.2)	
	G4	16 (5.5)	3 (2.9)	13 (7.0)		6 (4.2)	10 (6.8)	
Sarcomatoid differentiation	Absent	278 (95.9)	99 (95.2)	179 (96.2)	0.761^§^	137 (96.5)	141 (95.3)	0.770^§^
	Present	12 (4.1)	5 (4.8)	7 (3.8)		5 (3.5)	7 (4.7)	
Coagulative necrosis	Absent	248 (85.5)	88 (84.6)	160 (86.0)	0.732^#^	120 (84.5)	128 (86.5)	0.739^#^
	Present	42 (14.5)	16 (15.4)	26 (14.0)		22 (15.5)	20 (13.5)	
Microvascular invasion	Negative	252 (86.9)	90 (86.5)	162 (87.1)	1.000^#^	119 (83.8)	133 (89.9)	0.163^#^
	Positive	38 (13.1)	14 (13.5)	24 (12.9)		23 (16.2)	15 (10.1)	
Events								
Recurrence	No	250 (86.2)	96 (92.3)	154 (82.8)	0.032^#^	116 (81.7)	134 (90.5)	0.040^#^
	Yes	40 (13.8)	8 (7.7)	32 (17.2)		26 (18.3)	14 (9.5)	
Cancer-specific survival	Alive	269 (92.8)	100 (96.2)	169 (90.9)	0.105^§^	128 (90.1)	141 (95.3)	0.114^§^
	Dead	21 (7.2)	4 (3.8)	17 (9.1)		14 (9.9)	7 (4.7)	
Overall survival	Alive	236 (81.4)	87 (83.7)	149 (80.1)	0.530^#^	113 (79.6)	123 (83.1)	0.455^#^
	Dead	54 (18.6)	17 (16.3)	37 (19.9)		29 (20.4)	25 (16.9)	
Follow-up	Years (IQR)	7.85 (6.08–10.62)	8.95 (6.85–11.73)	7.11 (5.93–9.84)	<0.001^□^	7.50 (6.06–10.39)	8.18 (6.14–10.80)	0.429^□^

Abbreviations: eIF4E; eukaryonic translation initiation factor 4E, p-eIF4E; phospho-eIF4E, SD; standard distribution, IQR; interquantile range, ^#^; Chi-square test, ^§^; Fisher’s exact test, ^¶^; Student’s *t*-test, and ^□^; Mann-Whitney’s *U* test in comparison between groups with low and high expression of each protein. Values are presented as numbers (%) unless specifically indicated.

The potential clinical significance of eIF4E and p-eIF4E expression in the study cohorts is also presented in Table 1. eIF4E was expressed at significantly higher levels in patients with ccRCC recurrence than those without (*p =* 0.032). However, expression levels of eIF4E protein in ccRCC were comparable in different conditions of pT stage, lymph nodes, Fuhrman grade, sarcomatoid differentiation, coagulative necrosis, and microvascular invasion (MVI) (not significant for all). In contrast, peIF4E expression in ccRCC was significantly lower in the recurrent patients than in the recurrence-free patients (*p =* 0.040). The expression levels of p-eIF4E protein significantly differed in pT stage and pN (*p <* 0.001 and *p =* 0.030, respectively), whereas p-eIF4E protein was expressed at comparable levels in tumours of different Fuhrman grade, sarcomatoid differentiation, coagulative necrosis, and MVI. High eIF4E and low p-eIF4E expression was marginally associated with cancer-specific survival (CSS) (*p =* 0.105 and 0.114, respectively) but not with OS (Table 1).

**Figure 1:**
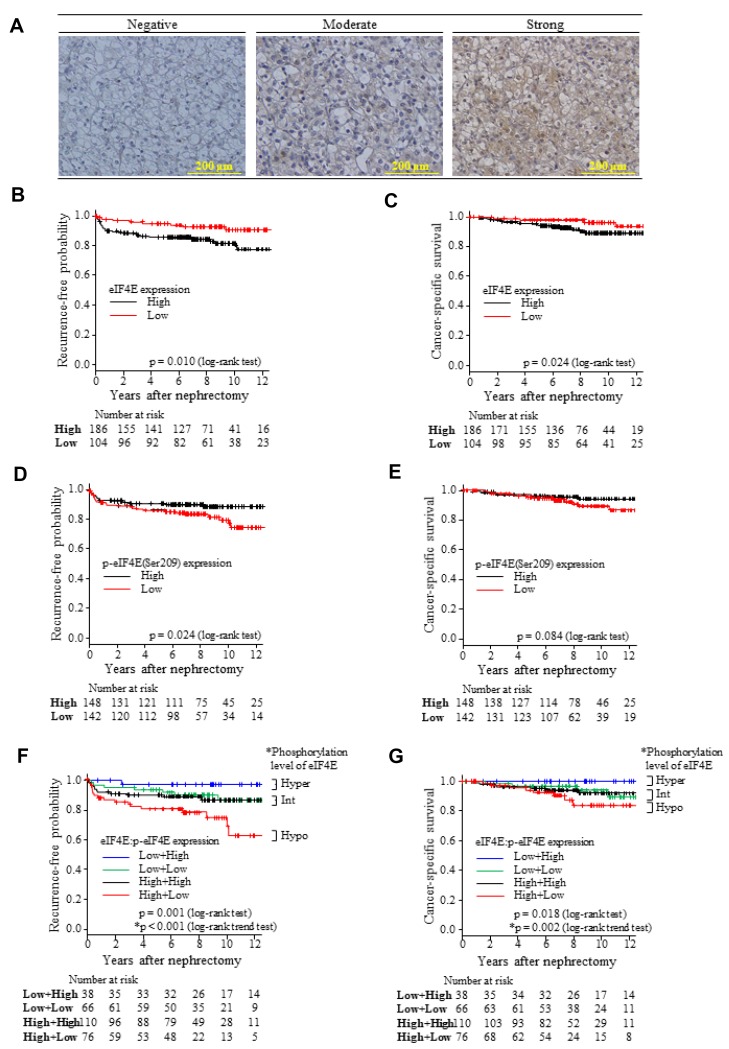
Grading of eIF4E and p-eIF4E(Ser209) expression levels by IHC semi-quantitation and their impact on recurrence-free and CSS intervals. (**A**) The representative panels of IHC intensity are shown. The panels were used to grade eIF4E and p-eIF4E expression in ccRCC specimens obtained by curative nephrectomy. The photos in these panels show p-eIF4E IHC (scale bars = 200 μm, original magnification 200×). IHC data were analysed as described in the Materials and Methods section. Kaplan-Meier curves for recurrence-free and CSS rates in the entire study cohort were stratified with high and low levels of eIF4E (**B**, **C**), p-eIF4E (**D**, **E**) expression, and a combination of eIF4E with p-eIF4E IHC stainability (**F**, **G**) in ccRCC tissues, respectively. The definition of phosphorylation levels of eIF4E (F, G) was presented in the Materials and Methods. ^*^The trends among hypo-, intermediate and hyper-phosphorylation levels of eIF4E were evaluated with log-rank trend tests (F, G).

In Kaplan–Meier survival analysis, the recurrence-free and CSS rates were significantly poorer as tumours progressed into advanced conditions of pT stages (pT1a, 1b, 2, 3 or 4), pN (pN0/X or 1), Fuhrman grade (1, 2, 3 or 4), sarcomatoid differentiation (absent or present), coagulative necrosis (absent or present), and MVI (positive or negative) for the entire cohort (*p <* 0.001 for each, respectively, log-rank test; Supplementary Tables 1 and 2, and Supplementary Figures 2 and 3). The recurrence-free and CSS intervals were significantly shorter in patients who presented with high eIF4E expression than those with low expression ((*p <* 0.05 for both, log-rank test; Figure 1B and 1C). In contrast, the recurrence-free interval (RFI) was significantly longer in patients who presented with high p-eIF4E expression than in those who did not (*p <* 0.05, log-rank test; Figure 1D), but Kaplan–Meier curves for CSS differed marginally between those with high and low peIF4E expression (*p =* 0.084, log-rank test; Figure 1E). In univariate and multivariate (model 1) Cox regression analyses (Table 2), pT1b≤ stage (vs. pT1a), Fuhrman grade 3/4 (vs. grade 1/2), presence of coagulative necrosis (vs. absence), and high eIF4E expression (vs. low) were significantly related to a high risk of recurrence and cancer-specific mortality. However, expression levels of p-eIF4E (high vs. low) were independent factors for predicting recurrence-free status but not for CSS (Table 2).

**Table 2:** Effects of clinicopathological factors on prognosis of ccRCC using Cox' proportional hazard analysis

Factors	Risk category		Univariate			Multivariate (model 1)			Multivariate (model 2)		
	1 (ref.)		HR	95% CI	*p*	HR	95% CI	*p*	HR	95% CI	*p*
Recurrence-free interval											
Pathological T stage	pTa	pT1b ≤	20.16	6.21–65.44	<0.001	8.46	2.47–29.02	<0.001	7.99	2.33–27.37	<0.001
Pathological N stage	pN0/X	pN1	8.13	2.50–26.45	<0.001	−	−	NS	−	−	NS
Fuhrman grade	G1/2	G3/4	7.84	4.17–14.71	<0.001	3.58	1.78–7.21	<0.001	3.47	1.71–7.04	<0.001
Sarcomatoid differentiation	Absent	Present	9.74	4.27–22.23	<0.001	−	−	NS	−	−	NS
Coagulative necrosis	Absent	Present	9.64	5.16–18.0	<0.001	4.22	2.11–8.44	<0.001	4.01	2.00–8.05	<0.001
Microvascular invasion	Negative	Positive	4.30	2.24–8.24	<0.001	−	−	NS	−	−	NS
eIF4E	Low	High	2.70	1.24–5.90	0.013	4.68	2.07–10.61	<0.001			
p-eIF4E(S209)	Low	High	0.48	0.25–0.92	0.027	0.47	0.24–0.92	0.029			
Phosphorylation levels of eIF4E#	Hypo	Int	0.41	0.22–0.76	0.005				0.38	0.20–0.75	0.005
	Hypo	Hyper	0.07	0.01–0.55	0.011				0.06	0.01–0.46	0.007
Cancer-specific survival											
Pathological T stage	pTa	pT1b ≤	28.49	3.82–212.70	0.001	9.91	1.21–81.28	0.033	9.81	1.19–80.61	0.034
Pathological N stage	pN0/X	pN1	10.92	2.51–47.51	0.001	−	−	NS	−	−	NS
Fuhrman grade	G1/2	G3/4	12.37	4.79–31.96	<0.001	4.27	1.53–11.93	0.006	3.88	1.37–10.99	0.011
Sarcomatoid differentiation	Absent	Present	6.28	1.82–21.72	0.004	−	−	NS	−	−	NS
Coagulative necrosis	Absent	Present	12.53	5.17–30.33	<0.001	4.17	1.56–11.16	0.005	4.21	1.55–11.43	0.005
Microvascular invasion	Negative	Positive	4.15	1.71–10.05	0.002	−	−	NS	−	−	NS
eIF4E	Low	High	3.40	1.11–10.36	0.032	3.66	1.21–11.04	0.021			
p-eIF4E(S209)	Low	High	0.46	0.18–1.14	0.092	−	−	NS			
Phosphorylation levels of eIF4E#	Hypo	Int	−	−	NS				−	−	NS
	Hypo	Hyper	−	−	NS				−	−	NS

Abbreviations: ccRCC; clear cell renal cell carcinoma, CI; confidential interval, HR; hazard ratio, ref.; reference, eIF4E; eukaryonic translation initiation factor 4E, p-eIF4E; phospho-eIF4E, NS: not significant. Hypo; hypophosphorylation, Int; intermediate phosphorylation, Hyper; hyperphosphorylation, #; Combinations of high eIF4E with low p-eIF4E, low eIF4E with high p-eIF4E or high eIF4E with low p-eIF4E, and low eIF4E with high p-eIF4E were defined as hypo-, intermediate, and hyper-phosphorylation levels of eIF4E, respectively.

The combination of eIF4E (high vs. low) and p-eIF4E (high vs. low) expression levels significantly stratified Kaplan–Meier curves for recurrence-free status and CSS after curative surgery (*p =* 0.001 and 0.018, log-rank test, respectively; Supplementary Figure 4). The stratification is considered to reflect differences in the phosphorylation levels of eIF4E (Figure 1F, 1G, and Supplementary Figure 4). RFI and CSS were significantly longer as eIF4E became more phosphorylated (*p =* 0.001 and *p =* 0.018 in log-rank test, *p <* 0.001 and *p =* 0.002 in log-rank trend test, respectively; Figure 1F, 1G, and Supplementary Figure 4). Univariate and multivariate (model 2) Cox analyses revealed that the phosphorylation levels of eIF4E would be an independent predictor of recurrence-free status, but not of CSS (Table 2).

### Protein and mRNA expression of eIF4E, p-eIF4E, and MNKs in cell lines

**Figure 2:**
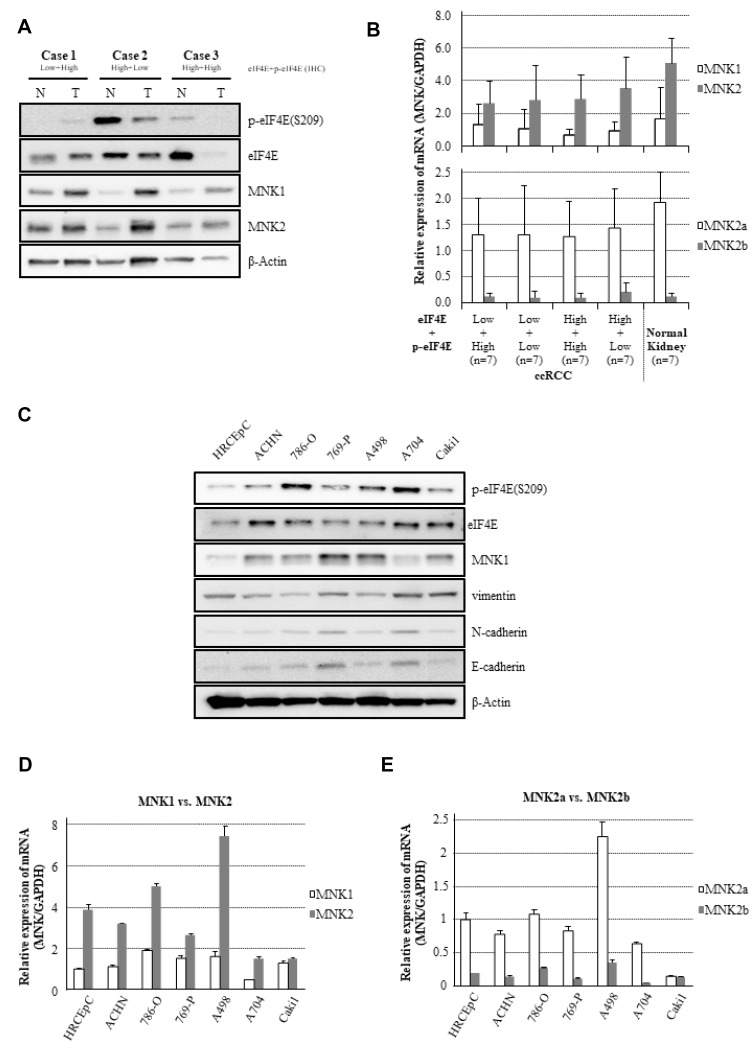
Expressional differences in MNK/eIF4E signaling and molecular markers on EMT. (**A**) Protein expression of eIF4E, p-eIF4E, MNK1, and MNK2 in ccRCC and the normal kidney on an immunoblot. Cases 1, 2, and 3 belonged to different groups stratified with a combination of eIF4E with p-eIF4E IHC stainability in Figure 1F and 1G. (**B**) Relative transcript levels of MNKs in ccRCC and the normal kidney tissues. (**C**) Protein expression of eIF4E, p-eIF4E, MNK1, and EMT markers in cell lines on an immunoblot. (**D**) Relative levels of MNK1 and MNK2 mRNAs in HRCEpC and RCC cell lines. (**E**) mRNA levels of MNK2a and MNK2b in HRCEpC and RCC cell lines. The experiments were repeated at least three times. Representative data are shown. Error bars show standard deviations. Abbreviations. N: the normal kidney parenchyma, T: tumour, HRCEpC: human renal cortical epithelial cell.

To the best of our knowledge, MNK1/2 are only physiological kinases to phosphorylate eIF4E *in vivo*, and the phosphorylated site is at Ser209 of eIF4E [9]. The expression levels of MNKs, eIF4E, and p-eIF4E (Ser209) were examined using fresh frozen tissue samples of ccRCC and normal kidney parenchyma from patients (*n =* 28 and 7, respectively). Cell lines were cultured, including a normal kidney cell line (human renal cortical epithelial cells: HRCEpC) and six human RCC cell lines (Figure 2C).

On the immunoblot, MNK1/2 was more expressed in ccRCC tissues than in normal kidney parenchyma, while the variable expression of eIF4E and p-eIF4E proteins was observed in ccRCC and the normal kidney (Figure 2A). At the mRNA level, MNK2, especially MNK2a, was a predominant isoform of the MNKs in ccRCC and normal kidney tissues (Figure 2B). No differences in transcript levels of MNK1, 2, 2a and 2b were found between the four categories stratified with eIF4E + p-eIF4E immunohistochemical (IHC) expression in ccRCC. Compared with HRCEpC, MNK1, eIF4E, and p-eIF4E proteins were more expressed in the RCC cell lines (Figure 2C). Expression profiles of MNK1 at mRNA and protein levels were similar among the cell lines except for A704, whereas MNK2 mRNA levels were greatly different among cell lines (Figure 2D and 2E). Variabilities of protein levels of MNK1:p-eIF4E expression were observed on the immunoblot, implicating the potential involvement of MNK2-dependent eIF4E phosphorylation (Figure 2C). Herein, in experiments with cell lines, we were unable to investigate MNK2, 2a, or 2b protein expression by western blotting due to the commercial unavailability of specific antibodies that are reliable for detecting MNK2s [10]. Instead, MNK mRNAs levels were quantified by reverse transcriptionpolymerase chain reaction (RTPCR) (Figure 2D and 2E). At transcript level, MNK2, especially MNK2a, was a predominant isoform of the kinases in HRCEpC and RCC cell lines. In Caki1, MNK2 and 2a mRNAs were expressed in amounts comparable to or more than those of MNK1 and 2b.

Taken together, eIF4E would be phosphorylated mainly by MNK2 (MNK2a) in ccRCC and the normal kidney parenchyma. In the following experiments, 786-O and A498 cell lines were used as typical ccRCC models with poorly and moderately differentiated features, respectively [11, 12].

### Effects of MNK inhibition on p-eIF4E and epithelial-mesenchymal transition (EMT) markers in 786-O and A498 cells

**Figure 3:**
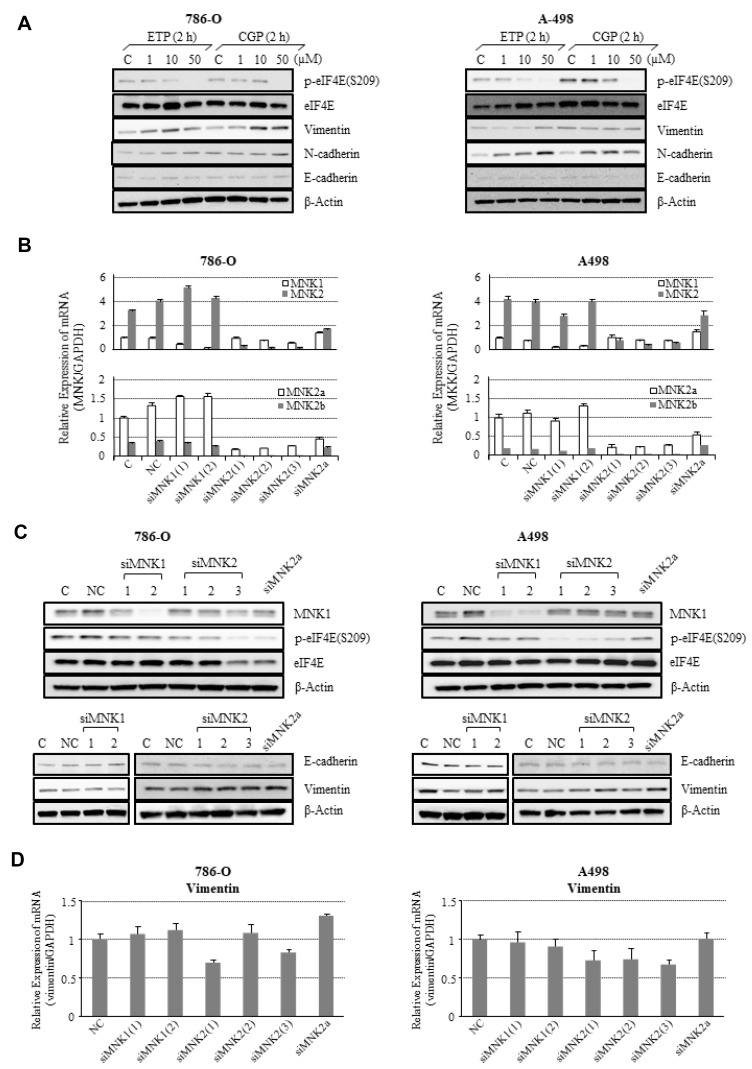
Effects of MNK inhibition on MNK/eIF4E signaling and expression of EMT markers in 786-O and A498 cells. (**A**) MNK1/2 were pharmacologically inhibited with a small-molecule agent, either CGP57380 or ETP45835 at concentrations of 0–50 μM for 2 h. (**B**) siRNAs specific to MNK1, MNK2, or MNK2a mRNAs successfully knocked down MNKs in 786-O and A498. Herein, the controls indicate conditions without any siRNA transfection. Each bar represents relative amounts of mRNA vs. MNK1 and MNK2a mRNA set as references, respectively. Error bars show standard deviations. (**C**) MNK2 and MNK2a knockdown lowered p-eIF4E expression, but MNK1 knockdown did not affect it significantly. Increase in vimentin and decrease in E-cadherin at protein levels in parallel with MNK2 or MNK2a knockdown, reflecting enhancement of EMT. (**D**) No significant increase in vimentin mRNA after knockdown of MNKs. Representative results are presented. The experiments were repeated at least three times. Abbreviations. C: control, NC: negative control.

We examined the influence of MNK inhibition on eIF4E phosphorylation in 786-O and A498 using a small-molecule MNK1/2 inhibitor CGP57380 (a classical agent) [9] or ETP45835 (recently developed) [13] (Figure 3A). In 786-O cells, CGP57380 and ETP45835 prevented eIF4E phosphorylation in a concentration-dependent manner and at the same time increased vimentin and N-cadherin levels (Figure 3A, left). Similarly, in A498 cells, p-eIF4E (vs. total eIF4E expression) was reduced in a concentrationdependent manner after CGP57380 or ETP45835 administration, with the reciprocal upregulation of vimentin and N-cadherin in parallel. E-cadherin appeared stable on the immunoblot, but this was hard to determine definitely because the bands of E-cadherin were too weak (Figure 3A, right).

Next, we attained sufficient knockdown of MNKs in 786-O and A498 using newly designed small interfering (si)-RNAs specific to MNK1, 2, or 2a mRNAs (Figure 3B). Under these conditions, MNK2 and 2a knockdown caused p-eIF4E reduction, although MNK1 knockdown had little effect on p-eIF4E expression (Figure 3C). In parallel, E-cadherin faintly decreased and vimentin became overexpressed at the protein level in 786-O and A498 cells with MNK2 or 2a knockdown (Figure 3C). Compared with RCC cells laden with a negative control (NC) of siRNA, however, no significant increase in vimentin mRNA was observed in the MNKs-knockdown cells, indicating that MNK knockdown may enhance translation of vimentin mRNA, but not its transcription (Figure 3D).

These findings suggest that MNK2a, but not MNK1, may phosphorylate eIF4E. They also suggest that MNK2a may suppress EMT in 786-O and A498 cells partially through negative regulation of vimentin at the translation level. However, the effects of siMNK2a on p-eIF4E were less than those of siMNK2 in A498 cells, implicating that MNK2b may be somewhat involved in phosphorylation of eIF4E depending on the cell type.

### Effects of MNK inhibition on cell migration, invasion and proliferation in 786-O and A498

**Figure 4:**
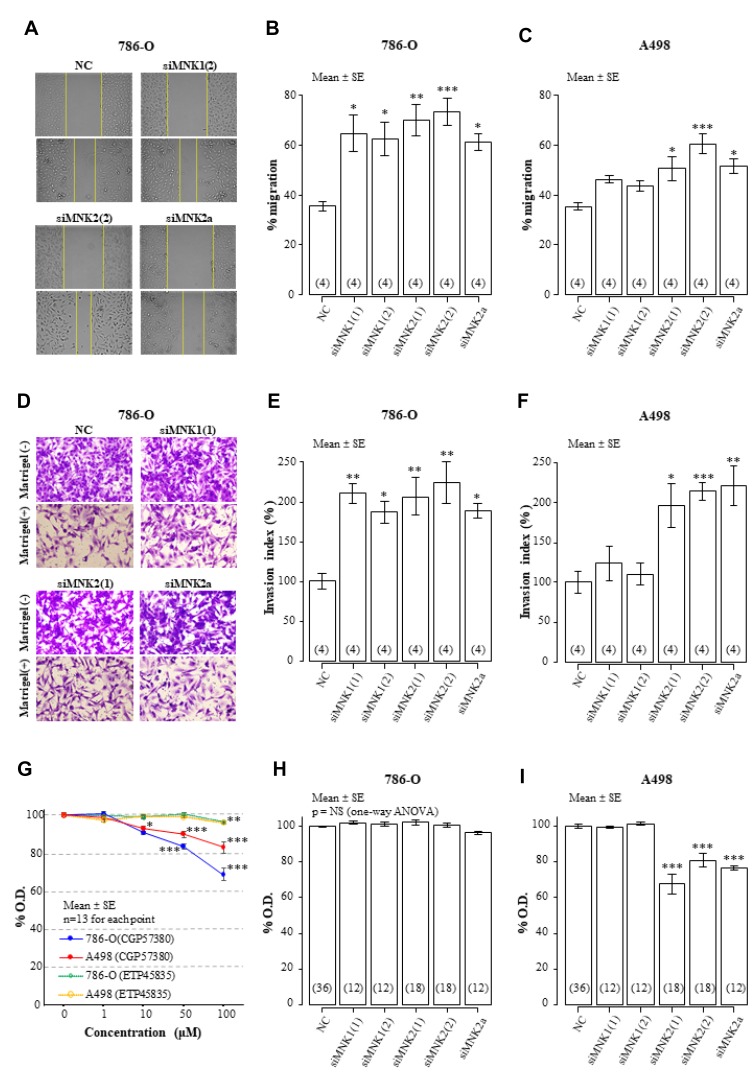
Effects of MNK inhibition on cell migration, invasion, and proliferation of 786-O and A498. (**A**–**C**) Scratch wound-healing assay under siRNA-treated conditions. (A) Representative actual images of 786-O migration for 7 h. Pooled data on the % migration of 786-O (B) and A498 cells (C) are presented as bar plots (*p* < 0.001 for each; one-way ANOVA). The data number is indicated in parenthesis at the bottom of each bar from four independent experiments. (**D**–**F**) Transwell invasion assay under siRNA-treated conditions. (D) Representative actual images of 786-O invasion through an 8 μm-sized porous membrane at 24 h in the absence and presence of Matrigel. Pooled data on the invasion index of 786-O (E) and A498 cells (F) are presented as bar plots (*p* = 0.001 and <0.001, respectively; one-way ANOVA). The data number is indicated in parenthesis at the bottom of each bar from four independent experiments (**G**–**I**). Cell viability was evaluated using an MTS assay under the conditions that MNKs were inhibited pharmacologically (G) or genetically with siRNAs (H and I) in 786-O and A498. The data number is indicated in parenthesis at the bottom of each bar from 6 independent experiments. Statistical significance was determined by one-way ANOVA followed by post-hoc test with Bonferroni adjustment; ^*^indicates *p* < 0.05; ^**^indicates < 0.01; ^***^indicates < 0.001 in multiple comparison tests with Bonferroni adjustment. Representative results are presented. Error bars show standard errors. Abbreviations. NC: negative control.

Figure 4A–4C shows the results of the scratch wound-healing assay for investigating the cell migration in MNKs-knockdown RCC cells. In 786-O and A498 cell lines, siMNK2 and siMNK2a significantly increased cell migration, compared with NC-siRNA. siMNK1 induced a remarkable increase in 786-O migration to the same extent as siMNK2 and siMNK2a. However, the enhancement by siMNK1 was not observed in A498. The Matrigel invasion assay demonstrated that siMNK2 and siMNK2a significantly enhanced cell invasion in 786-O and A498 (Figure 4D and 4F). In 786-O (but not in A498), knockdown with siMNK1 increased cell invasion approximately by twofold vs. the NC, comparable with that of siMNK2 or siMNK2a (Figure 4E and 4F).

The pharmacological inhibition of MNK1/2 with CGP57380 suppressed cell viability in a concentration-dependent manner, but the maximal effects at 100 μM CGP57380 were approximately a 30% and 10% decrease from the baselines in 786O and A498, respectively (Figure 4G). However, CGP57380 inhibits several kinases other than MNKs [14]. Another MNK1/2 inhibitor, ETP45835, had no effect on cell viability of 786-O and A498 at a concentration of 1 to 100 μM (Figure 4G). In 786-O cells treated with specific siRNAs against MNK1, 2, or 2a, cell viability was not altered at all (Figure 4H). In contrast, cell viability decreased by 30% in A498 cells with MNK2 or 2a knocked down, but not in those with MNK1 knocked down (Figure 4I).

MNK1/2 is related largely to cell migration and invasion rather than proliferation. MNK2a acts as a suppressor of cell migration and invasion, while MNK1 may be variably involved with migration and invasion, dependent on individual RCC cell lines.

## DISCUSSION

eIF4E expression and/or activity are increased in numerous cancers, playing a prooncogenic role [5, 6, 9, 10]. Consistent with a previous report [15], our results show that high eIF4E expression is independently prognostic of poor recurrence-free rate and CSS in the patients with localized ccRCC. However, the oncological relevance of peIF4E in RCC remains unclear. In the present study, we have demonstrated that higher expression of p-eIF4E is an independent predictor of longer RFI after curative nephrectomy for localized ccRCC. Cell migration and invasion are broadly regulated by EMT and are important processes in cancer metastasis [16]. The present data suggest that MNK2a-induced p-eIF4E may suppress EMT, cell migration, and invasion, partially due to vimentin downregulation at the translational level, consequently inhibiting ccRCC recurrence.

Significant correlations between p-eIF4E expression and poor prognosis have been reported on various tumours [17–21]. However, in the present study, p-eIF4E may associate with the negative regulation of eIF4E activity, as indicated in some other tumours including ovarian, gastric, and colorectal cancers [22, 23]. MNK1/2 kinases are more expressed in RCC than in the normal kidney tissue and HRCEpC. MNK2a, a predominant isoform of the MNKs in RCC, may work as a central player in eIF4E phosphorylation to regulate EMT and metastasis negatively in localized ccRCC. These findings strongly favour the clinical relation between higher (or lower) peIF4E expression and longer (or shorter) RFS of patients with localized ccRCC, respectively.

MNK2a can be negatively controlled through its phosphorylation at Ser437 by mTORC1 [24]. Conversely, MNK2 selectively interacts with mTORC1 in a kinaseindependent manner and inhibits S6K phosphorylation [25]. Recently, we demonstrated that the lower or higher expression of eIF4E reflected a weaker or stronger activation of the mTORC1/4EBP1/eIF4E signalling pathway, respectively [26]. In the present study, significantly lower eIF4E and higher p-eIF4E expressions were observed in ccRCC patients who did not experience tumour recurrence than in those who did. In the patients with better prognosis, hypofunction of mTORC1 may suppress mTORC1/4EBP1/eIF4E signalling to reduce eIF4E release from 4EBP1 [5, 9], and simultaneously may stimulate MNK2a to boost up eIF4E phosphorylation [24].

**Figure 5:**
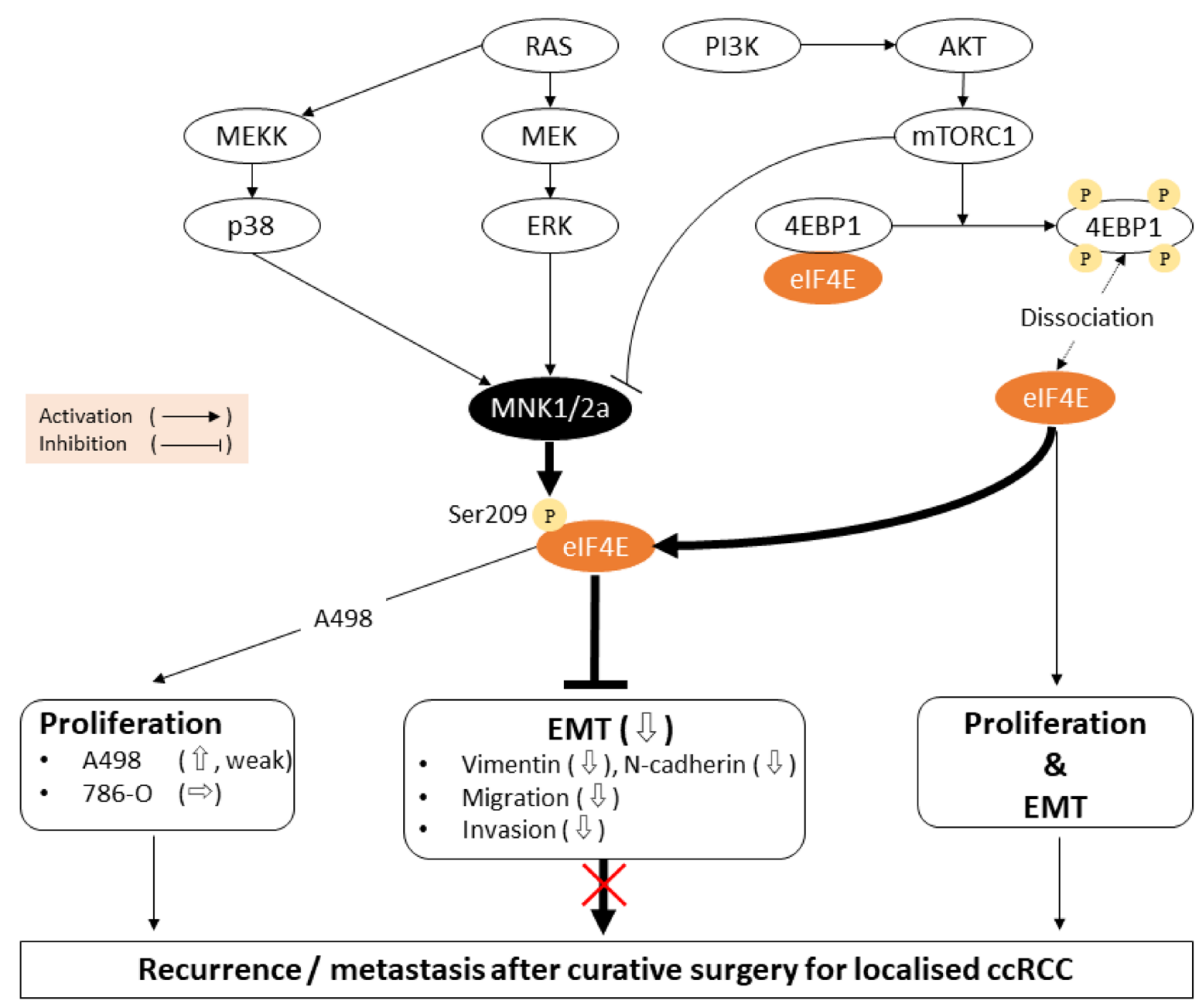
Schematic diagrams of the signaling pathways and biological reactions in this study. The MNK/eIF4E axis is located at the downstream of two major intracellular signalling, mTORC1/4EBP1/eIF4E and Ras/MEK/extracellular signal-regulated kinase pathways (ERK). MNK2a may predominantly phosphorylate eIF4E due to the higher expression than MNK1 and MNK2b in ccRCC. MNK2a-induced p-eIF4E inhibits the biosynthesis of EMT-related proteins (e.g., vimentin), and suppresses cell migration and invasion, leading to the decrease in metastatic recurrence of ccRCC after curative nephrectomy. MNK2a is weakly involved in cell proliferation in A498 cells. The mechanism that MNK1 inhibits 786-O migration and invasion has been unresolved. Downward, upward, and horizontal open arrows represent a decrease, an increase, and no change, respectively.

The molecular hallmarks of EMT are canonically the decrease in epithelial traits, including E-cadherin and the increase in mesenchymal features such as vimentin and N-cadherin, leading to facilitated motility, plasticity, and stemness of tumour cells [16, 27]. Vimentin expression could predict poor clinical outcomes of RCC [28, 29], independently of grade and stage [30, 31]. In the present study, MNK2 or 2a inhibition augmented migration, invasion and vimentin expression to promote EMT. In contrast, in pancreatic, breast and lingual cancer cells, MNK inhibition reduces vimentin protein levels and reverses EMT [32, 33]. Maimon *et al*. demonstrated that MNK2a and 2b play anti- and prooncogenic roles with an opposing function, respectively, suggesting that a balanced level of their antagonism could determine a whole nature of a tumour [34]. In triple-negative breast cancer cells that predominantly express MNK1, MNK1b overexpression with gene transfection facilitates cell migration and invasion [35]. The overwhelming antagonism of abundant MNK2a against MNK2b in ccRCC may suppress EMT along with p-eIF4E upregulation (Figure 5). To our speculation, the antagonistic balance between MNKa and b in a major MNK1/2 isoform could possibly control EMT progressively or reversely.

Relationships among MNK activity, p-eIF4E and proliferation have not been fully resolved [10]. Neither MNK activity nor p-eIF4E was required for normal cell proliferation [36, 37]. In breast cancer cells overexpressing MNK1a or 1b with gene transfection, cell viability was increased or remained stable depending on the cell lines [35]. Colony formation was greatly impaired in mesenchymal embryonic fibroblasts expressing eIF4E with mutation of Ser209 to alanine which MNKs cannot phosphorylate, but the fibroblasts proliferated comparably as those with wildtype eIF4E [21]. Our results show that MNK2/2a inhibition supressed cell proliferation by 20–25% in A498 cells but did not in 786-O cells. These data support the remark that the MNK/eIF4E axis is not tightly connected to tumour proliferation and growth [10].

Our study has some limitations. Firstly, this study was retrospective at a single centre, without consideration of prognostic factors, such as hypertension, obesity and smoking habits [38]. Secondly, molecular subtyping of ccRCC was not performed [39, 40]. Thirdly, ccRCC heterogeneity in gene expression and intracellular signalling was not considered [41]. In the present study, eIF4E and p-eIF4E expression on immunoblot was partially inconsistent with IHC classification of eIF4E phosphorylation levels. The discrepancy may result from the heterogeneity within a tumour. Fourthly, it was not possible to investigate the effects of specific MNK inhibition on p-eIF4E in mouse xenograft models, because stable MNK knockdown in RCC cell lines with short hairpin RNA significantly decreased eIF4E protein expression under our experimental conditions. Further studies are needed to confirm the present findings.

In conclusion, lower eIF4E and higher p-eIF4E expression is an independent predictor of longer RFI after curative nephrectomy for localized ccRCC. MNK2a may predominantly phosphorylate eIF4E due to the higher expression than MNK1 and MNK2b in ccRCC. MNK2a-induced phosphorylation of eIF4E inhibits the biosynthesis of EMT-related proteins and ultimately suppresses metastatic recurrence of ccRCC. The present data suggest that the activity of MNK2a/eIF4E axis may provide a rationale for stratifying ccRCC patients at risk of recurrence and a therapeutic strategy for ccRCC.

## MATERIALS AND METHODS

### Reagents and antibodies

MNK inhibitors, CGP57380 (cat. No; 13322) and ETP45835 (cat. No; 5183) were purchased from Cayman Chemical (Ann Arbor, MI, USA) and Tocris Bioscience (Bristol, UK), respectively. For immunoblot and immunohistochemistry (IHC), the antibodies to eIF4E (cat. No; 2067) and MNK1 (cat. No; 2195) were bought from Cell Signaling Technology Japan (Osaka, Japan). Antibodies to vimentin (cat. No; 550513), N-cadherin (cat. No; 610920), and E-cadherin (cat. No; 610181) were bought from BD Bioscience (Franklin Lakes, NJ, USA). Anti-β-actin (cat. No; ab49900) and p-eIF4E (Ser209) (cat. No; ab76256) antibodies were obtained from Abcam Inc. (Cambridge, MA, USA). An antibody to MNK2 (cat. No; 17354-1-AP) was purchased from Proteintech Japan (Tokyo, Japan).

### Collection and acquisition of patients’ clinical data and tumour specimens

We retrospectively explored medical archives of consecutive patients who underwent curative surgery for localized ccRCC in Yamagata University Hospital from 1997 to 2011. Patients with genetic disorders predisposing ccRCC (such as VHL disease, distant metastasis, synchronous bilateral RCC), or those undergoing neoadjuvant or adjuvant anti-tumour chemotherapy with molecular-targeted drugs, or those with preoperative arterial embolisation were excluded from the present study. For inclusion criteria, patients were pathologically diagnosed with ccRCC, and the surgical specimens were available as formalin-fixed paraffin-embedded blocks. Finally, 290 patients with localized ccRCC were eligible for the present study. Clinical and pathological information on the patients were collected from medical records.

### Pathology, immunohistochemistry and semi-quantitative evaluation

#### Pathologic examination

All specimens surgically obtained were fixed in 10% buffered formalin and embedded in paraffin blocks, and were diagnosed by staff pathologists according to the routine procedures in the institution. T classification and pathological findings were re-assessed for the present study, according to the World Health Organization classification 2004 [42], and the 2010 American Joint Committee on cancer TNM staging system (T.K., M.Y.) [43]. A single representative block was selected for each patient.

### Immunohistochemistry

The IHC staining against eIF4E and p-eIF4E (Ser209) was carried out with a standard procedure described elsewhere [26]. The detailed procedures were provided as part of Supplementary Materials and Methods.

### Semi-quantitative evaluation and grading of eIF4E phosphorylation levels

Immunohistochemically stained sections were evaluated with a semi-quantitative method as described elsewhere [26]. The expression of the proteins detected by IHC was scored with the mean area and staining intensity of positive tumour cells on an Olympus BX50 microscope (Olympus Corporation, Tokyo, Japan). Brown-coloured staining of tumour cells, regardless of the cytoplasmic or nuclear stainability, was judged as positive for anti- eIF4E and p-eIF4E antibodies. The mean area of staining cells was evaluated as staining area ≤50 (score = −) or >50 % (score = 1+) of tumour cells. The mean staining intensity was graded as negative (score = −), moderate (score = 1+), and strong (score = 2+) (Figure 1A). The semi-quantitative evaluation of IHC slides was made by two observers (O.I, and S.N.) independently without the knowledge of the clinical outcomes of the corresponding patients. If there was any discrepancy between the observers, they re-analysed the slides together and made a consensus of the final evaluation. The final scores were obtained through the addition of area and intensity scores. High expression was defined as a strong positive intensity with >50% staining area (final scores = 3+), and patients with final scores ≤2+ were defined as having low protein expression. Phosphorylation levels of eIF4E was graded as hypo, intermediate, and hyper -phosphorylation, based on a combination of eIF4E and p-eIF4E expression: Combinations of high eIF4E with low p-eIF4E, and low eIF4E with high p-eIF4E indicated hypo and hyper-phosphorylation levels of eIF4E, respectively. Low eIF4E plus low p-eIF4E or high eIF4E plus high p-eIF4E were categorised as the intermediate phosphorylation level.

### Preparation of ccRCC and normal human kidney tissues

Fresh frozen tissue samples obtained from patients with RCC (*n =* 28) who underwent nephrectomy at Yamagata University Hospital were used in the present study. Each of the tumour specimens came from a sample per patient. Non-tumourous renal parenchymal specimens away from RCC areas (*n =* 7) were obtained from different 7 patients of the 28 cases. Samples cut from ccRCC tissues as well as samples of non-tumorous kidney parenchyma were freshly frozen and maintained at −80° C during storage.

### Cell culture

The RCC cell lines ACHN, 786-O, 769-P, A498, A704, and Caki1 were obtained from the American Type Culture Collection (Manassas, VA, USA). Cells were cultured in RPMI medium supplemented with 50 μg/mL of kanamycin and 10% foetal bovine serum in an incubator at 5% CO_2_ and 37° C. HRCEpC was obtained from PromoCell GmbH (Heidelberg, Germany). Cells were cultured in Renal Epithelial Cell Growth Medium 2 (PromoCell GmbH) including growth supplements in an incubator at 5% CO_2_ and 37° C. In most experiments, 786-O (mutated in *VHL* and *PTEN*) and A498 (mutated in *SETD2* and often *VHL*, and high HIF2α-laden) cell lines were used as ccRCC models with poorly and moderately differentiated features, respectively [11, 12].

### Immunoblot analysis

The immunoblot analysis was performed as described previously [44], using SuperSignal West Pico Substrate (Pierce, Rockford, IL, USA) and Western BLoT Hyper HRP Substrate (Takara Bio Inc., Shiga, Japan) according to the manufacturers’ instructions. Images were analysed using UN-SCAN-Itgel Automated Digitizing System software (Version 5.1 for Windows, Silk Scientific Inc., Orem, UT, USA). The antibodies to the following chemicals were used: eIF4E, p-eIF4E (Ser209), MNK1, MNK2, vimentin, N-cadherin, and E-cadherin. β-actin was used as a loading control.

### Quantitative RT-PCR (qRT-PCR)

The general procedures for qRT-PCR were provided as part of Supplementary Materials and Methods. MNK1, 2, and 2a mRNAs were quantified using qRT-PCR and their amounts were compared among RCC cell lines. Sequences of the primers used are shown in Supplementary Figure 1.

### siRNA transfection

For MNKs silencing, 786-O and A498 cells were transfected with specific human siRNAs against MNK1 (1 or 10 nM), MNK2 (5 or 10 nM), or MNK2a (5 or 10 nM) by using Lipofectamine RNAiMAX (Invitrogen, Thermo Fisher Scientific Inc.) according to the manufacture’s recommendations. Targeting sequences of MNKs siRNA are as follows: siMNK11 (#161707029), 5′-GCCGUCAAAAUCAUCGAGAAACAAG-3′; siMNK12 (#161707032), 5′-GUUUACAGAUGGUAUCUUCUCAAAA-3′; siMNK21 (#158253115), 5′-GAAGUUUUCCUUUACACCAACUGTC-3′; siMNK22 (#158253118), 5′-GCCUUGGACUUUCUGCAUAACAAAG-3′; siMNK22 (#165395347), 5′-AGUGCAGACCUGCAUCAACCUCATC-3′; siMNK2a (#165395350), 5′-CUUGUCCCCAUGAUAGUUGACAATC-3′ (all from Integrated DNA Technologies, Inc. Tokyo, Japan). Non-specific control siRNA (Integrated DNA Technologies, Inc.) was used as NC.

### Scratch wound-healing assays

Six-well culture dishes containing a single layer of either 786-O or A498 cells were treated with siRNA transfection then immersed in 2ml growth medium.

Afterwards, they were scratched using a pipette tip to form a wound across the diameter. After being washed twice with phosphate buffered saline to remove any debris, cells were incubated in the media at 37° C for 7 h, and could migrate into the wound for the period of time. At 0h and 7h, a BZ-X700 microscope (Keyence Co. Osaka, Japan) was used to take images (×10 objective lens). Wound healing was assessed by tracing the borders of the wound. The average percentage of cell-free area after cell culture compared to the initial area was calculated using the Image J software (version 1.50, https://imagej.nih.gov/ij/index.html).

### Cell invasion assay

A cell invasion assay was performed according to manufacturer’s instructions (https://www.corning.com). Cells (1.0 × 10^5^ per well) transfected with siRNA against MNKs or NC were seeded on top of the Matrigel-coated insert with 8 μm pores in serum-free RPMI medium in the upper chambers (Corning BioCoat Matrigel Invasion Chamber; # 354480, Corning International, Tokyo, Japan). The medium supplied with 10% foetal bovine serum was used in the lower chamber. After 24 h incubation, the cells on top of the inserts were scraped off using a cotton swab. The cells at the bottom of the inserts were fixed and stained with crystal violet. Cell images were captured on an Olympus BX43 microscope equipped with an Olympus DP21 camera and were analysed for cell numbers with the Image J software. Invaded cells were counted under × 200 magnification in at least five representative fields and the mean for each chamber was determined. The %invasion is defined as the percentage of cells having crossed the porous membrane of the chambers in the presence of a layer of Matrigel versus in its absence. The invasion index was calculated as follows: (%invasion in the experimental group / %invasion in the control group) × 100. The experiments were repeated at least three times.

### Cell proliferation assay

786-O and A498 RCC cells were cultured at 72 h in the presence and absence of MNK1/2 inhibitors, CGP57380 or ETP45835. The cells transfected with siRNA against MNKs or NC were cultured at 48h. Cell viability was estimated as %O.D. values using CellTiter 96 Aqueous One Solution Cell Proliferation Assay (Promega) as described previously [44].

### Statistical analyses

Comparisons between two categories were performed using cross tabulation and Chisquare or Fisher’s exact tests. A Kaplan–Meier survival curve analysis and a logrank test were used to estimate and compare postoperative intervals of recurrencefree states and CSS between two groups. When the overall test result was statistically significant, a log-rank trend test was performed in case of examining a tendency among three groups. The RFI was defined as the duration from the treatment initiation (curative surgery) to the first appearance of a metastasis or local emergence of tumours at, or adjacent to, the primary site. The CSS interval was defined as the duration of follow-up calculated from the date of surgery to the date of death or last follow-up. The variables that significantly contributed to prognosis by univariate analyses were investigated by multivariate analysis. The prognostic variables in predicting recurrence-free and CSS rates were assessed by univariate and multivariate Cox proportional hazards analysis. A stepwise regression method was carried out with a significance level of 0.05 for variable entering.

Continuous variables are presented as the median or the mean ± standard deviation (SD) or standard error (SE). They were statistically analysed using MannWhitney’s U test, *t*test, analysis of variance (ANOVA) and, if necessary, a posthoc Bonferroni test for multiple comparisons. All pvalues were based on the twosided statistical analysis. *p <* 0.05 was considered statistically significant. All analyses were performed using R statistical software version 3.4.1 (http://cran.rproject.org/).

### Ethical approval

The present study was carried out in accordance with a protocol approved by the Ethical Committee of Yamagata University School of Medicine (No. 534 approved on March 2, 2018). The ethical boards waived the requirement for individual informed consent because the present study was retrospective, and the anonymity of the participants was ensured. This study has been performed in accordance with the ethical standards as laid down in the 1964 Declaration of Helsinki and its later amendments or comparable ethical standards.

## SUPPLEMENTARY MATERIALS







## Abbreviations

ccRCC: clear cell renal cell carcinoma
CSS: cancer-specific survival
4EBP1: eIF4Ebinding protein 1
eIF4E: eukaryotic initiation factor 4E
EMT: epithelialmesenchymal transition
HIF: hypoxia-inducible factor
HRCEpC: human renal cortical epithelial cells
MNK: MAP kinase interacting kinase
mTORC1: mammalian target of rapamycin complex 1
MVI: microvascular invasion
NC: negative control
OS: overall survival
RFI: recurrence-free interval
SETD2: Set domain containing 2
siRNA: small interfering siRNA
VHL: von Hippel-Lindau
