# Supplementary material for: Impact of eIF4E phosphorylation at Ser209 via MNK2a on tumour recurrence after curative surgery in localized clear cell renal cell carcinoma
Source: Oncotarget. 2019 Jun 18;10(40):4053–68. doi: 10.18632/oncotarget.27017 (PMC6592294; doi:10.18632/oncotarget.27017)
Supplement: Supplementary file 1 [file oncotarget-10-4053-s001.pdf]

Impact of eIF4E phosphorylation at Ser209 via MNK2a on tumour recurrence after curative surgery in localized clear cell renal cell carcinoma

SUPPLEMENTARY MATERIALS

Pathology, immunohistochemistry, and semi-quantitative evaluation

Immunohistochemistry

The immunohistochemical staining against eIF4E and p-eIF4E (Ser209) was carried out using a standard procedure described elsewhere. Tissue sections (3 µm) obtained from the formalin-fixed paraffin-embedded specimens were deparaffinised in xylene and rehydrated. Endogenous peroxidase was blocked with 3% H_2O_2 in methanol for 15 min. Sections were incubated with primary antibodies overnight at 4° C in a moist chamber after epitopes were reactivated by autoclaving the sections in 10 mM citric buffer (pH 6.0) for eIF4E and in 10 mM Tris-EDTA (pH 9.0) for p-eIF4E at 120° C for 20 min. Detection was performed via the peroxidase method, using 3,3′diaminobenzidine and H_2O_2. The sections were counterstained with haematoxylin for nuclear detection, dehydrated, and mounted as permanent histological specimens. Negative controls consisted of sections where the primary antibody was replaced with non-immune rabbit serum. Positive controls for the antigens' immunoreactivity consisted of human breast cancer tissue (data not shown).

Quantitative reverse transcription-polymerase chain reaction (qRT-PCR)

MNK1, 2, and 2a mRNAs were quantified using qRT-PCR and their amounts were compared among RCC cell lines. Total cellular RNA was extracted using the SV total RNA Isolation System (Promega, Madison, WI, USA) for cell lines and using the mirVana miRNA isolation kit (Themo Fisher Scientific Inc., Yokohama, Japan) for specimens; the first-strand DNA was synthesized using a cDNA Reverse Transcription kit (Applied Biosystems, Thermo Fisher Scientific Inc.) according to the manufacturer's instructions. Real-time qRT-PCR was performed in the 7300 Real-time PCR System (Applied Biosystems, Thermo Fisher Scientific Inc.).

qRT-PCR primers in this study were specifically designed with reference to Primer-BLAST (https// www.ncbi.nlm.njh.gov/tools/primer-blast/). Sequences of the primers used are shown in Supplementary Figure 1. GAPDH was used as an endogenous control, sequences of the following primers: Forward 5′-GCACCGTCAAGGCTGAGAAC-3′, Reverse 5′-TGGTGAAGACGCCAGTGGA-3′. The experiment was conducted in in triplicate wells for each sample, according to the standard protocol and using a TB Green Premix Ex Taq II (Takara Bio Inc., Shiga, Japan) and repeated at least three times to confirm reproducibility. Relative mRNA expression was determined via the ΔΔCT method with CT values, corresponding to the PCR cycle number at which fluorescence emission reaches a threshold above baseline emission, determined for mRNA expression relative to that of untreated controls.

Sequences of qRT-PCR primers for MNK

Primer	Sequence
MNK1_F	5'- TGCTTGGAGAGGGAGCCTAT -3'
MNK1_R	5'- TGCCCTGCTTGTTTCTCGAT -3'
MNK2_F	5'- GCAGACCTGCATCAACCTGA -3'
MNK2_R	5'- CCTGAAAACCCTGCTCCGAA -3'
MNK2a/2b_F1	5'- CACCTTGCCCACTCCCAT -3'
MNK2a_R1	5'- GTGAGGTAGCTCGGACCA -3'
MNK2b_R2	5'- GAGGAAGTGACTGTCCCAC -3'

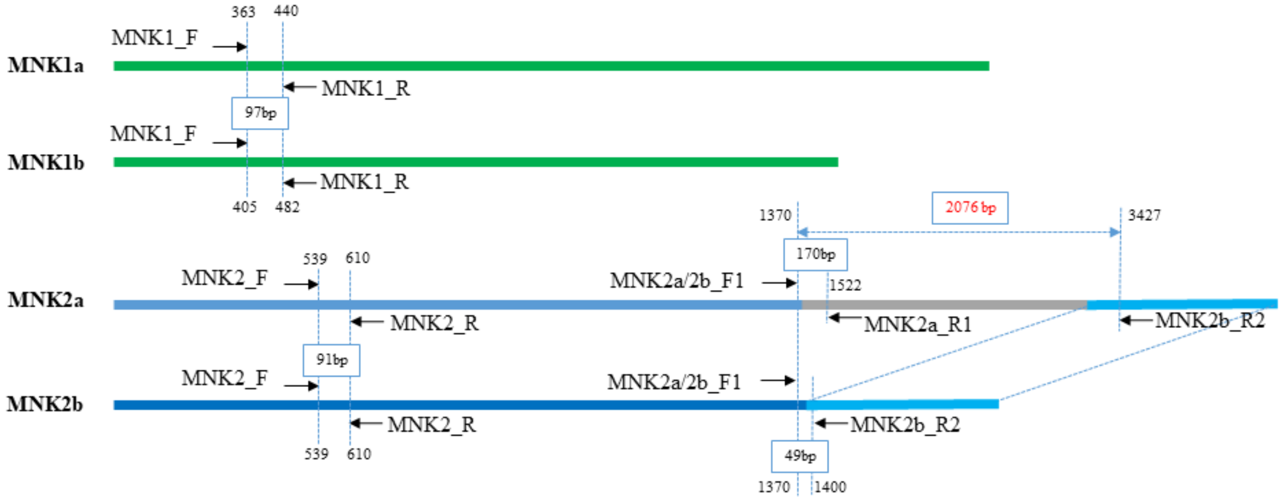

Supplementary Figure 1: Sequences of qRT-PCR primers for MNKs. Abbreviations. qRT-PCR; quantitative reverse transcription-polymerase chain reaction, F; forward, R; reverse.

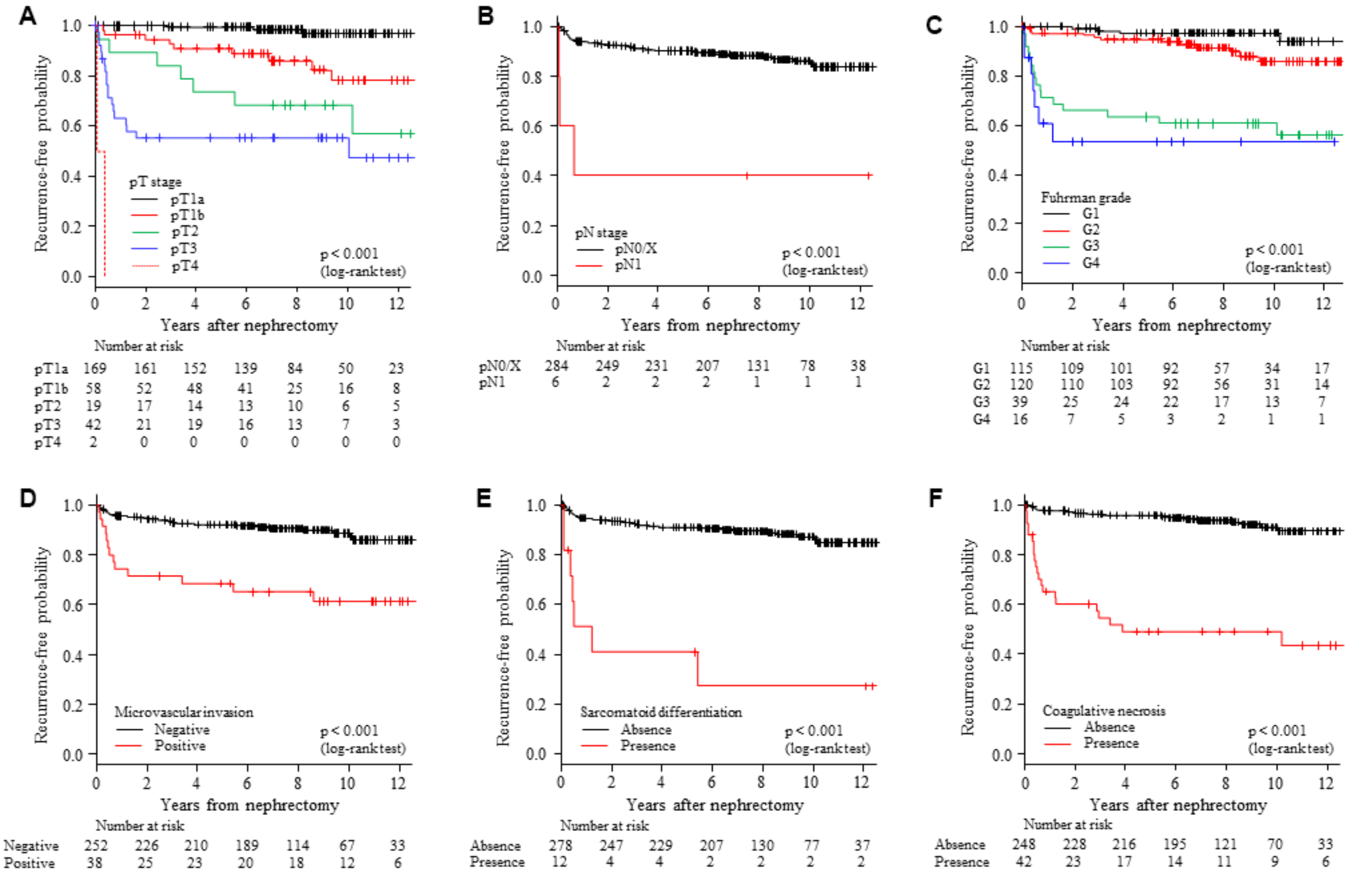

Supplementary Figure 2: Effects of clinicopathological factors on recurrence-free probability for the entire cohort. (**A**) pathological T (pT) stage, (**B**) pathological N (pN) stage, (**C**) Fuhrman grade, (**D**) microvascular invasion, (**E**) sarcomatoid differentiation, and (**F**) coagulative necrosis.

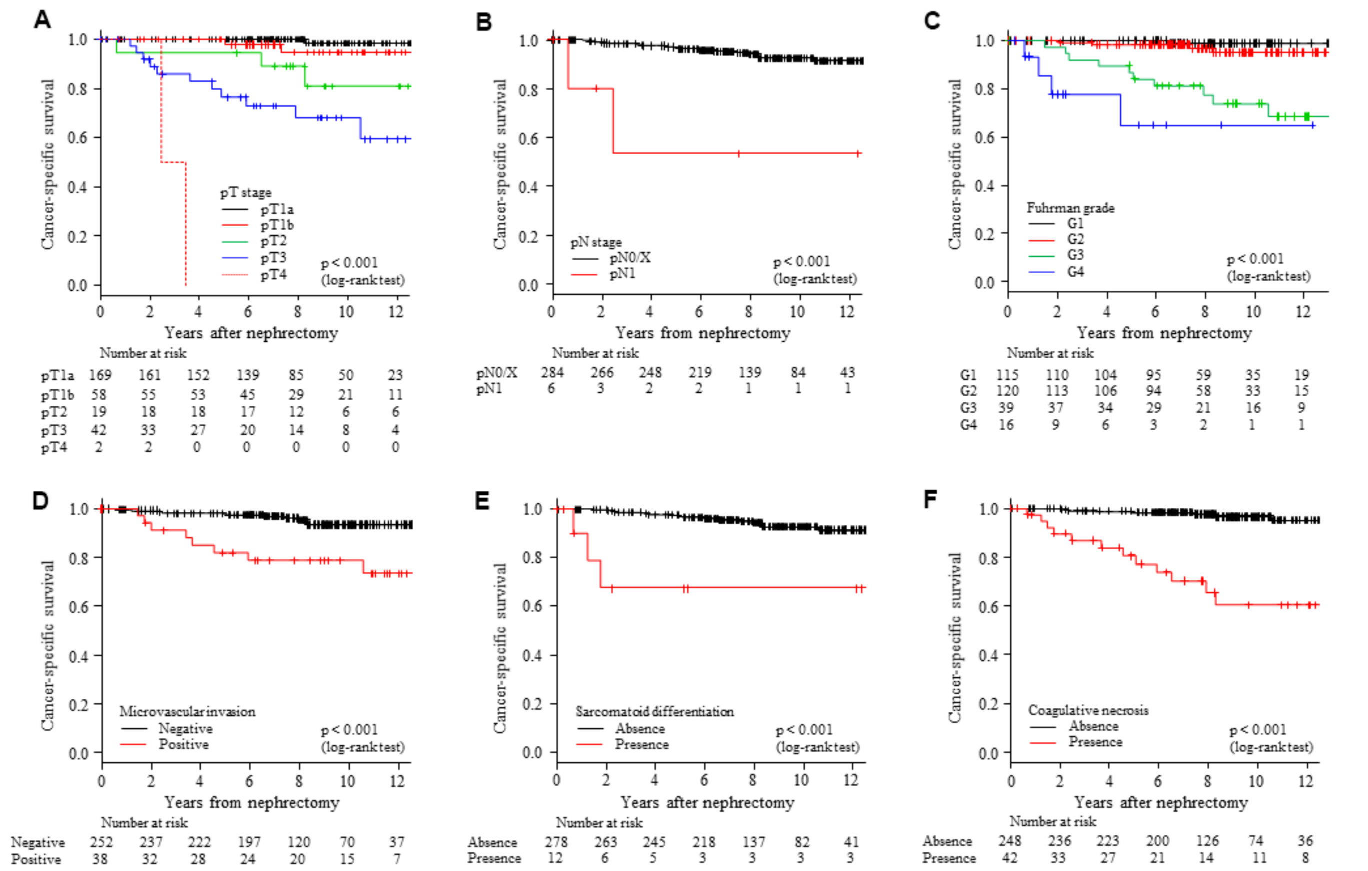

Supplementary Figure 3: Effects of clinicopathological factors on cancer-specific survival for the entire cohort. (**A**) pathological T (pT) stage, (**B**) pathological N (pN) stage, (**C**) Fuhrman grade, (**D**) microvascular invasion, (**E**) sarcomatoid differentiation, and (**F**) coagulative necrosis.

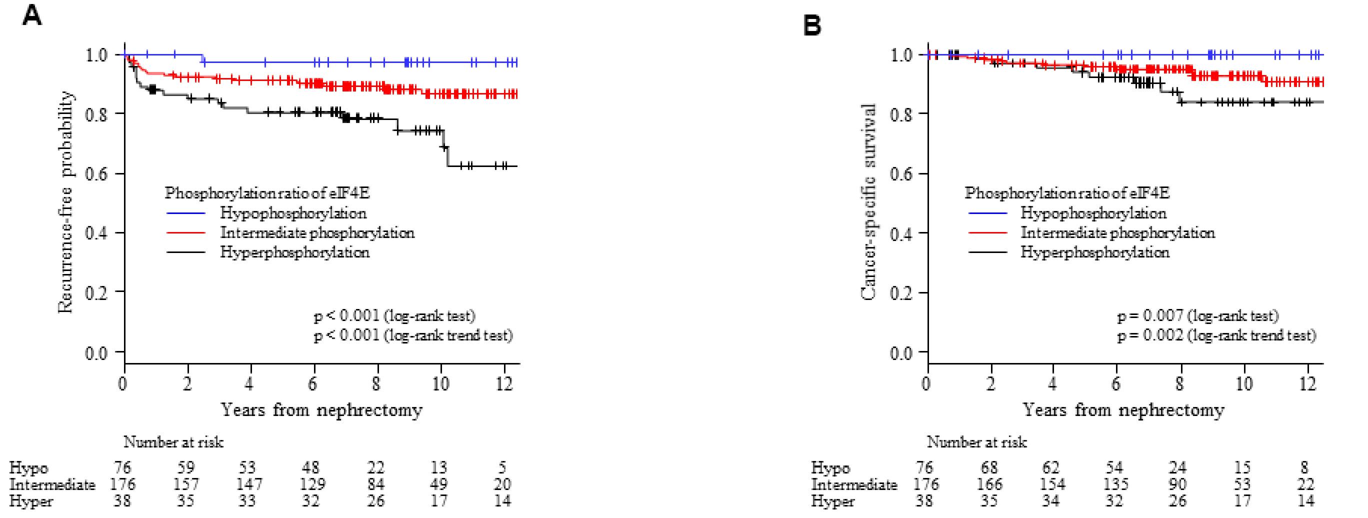

Supplementary Figure 4: Effects of eIF4E phosphorylation ratio in ccRCC tissues on recurrence-free status and CSS for the entire cohort. The probabilities of recurrence-free status (**A**) and CSS (**B**) became significantly poorer as eIF4E in ccRCC tissues became less phosphorylated.

Supplementary Table 1: Estimated recurrence-free rates of the study patients analyzed with Kaplan-Meier method. See Supplmentary Table 1

Supplementary Table 2: Cancer-specific survival of the study patients analyzed with Kaplan-Meier method. See Supplmentary Table 2
